# Thiol Redox Sensitivity of Two Key Enzymes of Heme Biosynthesis and Pentose Phosphate Pathways: Uroporphyrinogen Decarboxylase and Transketolase

**DOI:** 10.1155/2013/932472

**Published:** 2013-07-16

**Authors:** Brian McDonagh, José Rafael Pedrajas, C. Alicia Padilla, José Antonio Bárcena

**Affiliations:** ^1^Department of Biochemistry and Molecular Biology, University of Córdoba and Córdoba Maimónides Institute for Biomedical Research (IMIBIC), 14071 Córdoba, Spain; ^2^Department of Musculoskeletal Biology, Institute of Ageing and Chronic Disease (IACD), University of Liverpool, Liverpool L69 3GA, UK; ^3^Molecular Signaling and Antioxidant Systems in Plants, Department of Experimental Biology, University of Jaén, 23071 Jaén, Spain

## Abstract

Uroporphyrinogen decarboxylase (Hem12p) and transketolase (Tkl1p) are key mediators of two critical processes within the cell, heme biosynthesis, and the nonoxidative part of the pentose phosphate pathway (PPP). The redox properties of both Hem12p and Tkl1p from *Saccharomyces cerevisiae* were investigated using proteomic techniques (SRM and label-free quantification) and biochemical assays in cell extracts and *in vitro* with recombinant proteins. The *in vivo* analysis revealed an increase in oxidized Cys-peptides in the absence of Grx2p, and also after treatment with H_2_O_2_ in the case of Tkl1p, without corresponding changes in total protein, demonstrating a true redox response. Out of three detectable Cys residues in Hem12p, only the conserved residue Cys52 could be modified by glutathione and efficiently deglutathionylated by Grx2p, suggesting a possible redox control mechanism for heme biosynthesis. On the other hand, Tkl1p activity was sensitive to thiol redox modification and although Cys622 could be glutathionylated to a limited extent, it was not a natural substrate of Grx2p. The human orthologues of both enzymes have been involved in certain cancers and possess Cys residues equivalent to those identified as redox sensitive in yeast. The possible implication for redox regulation in the context of tumour progression is put forward.

## 1. Introduction

During the initiation and progression of any disease state there is a shift in the metabolic programming within the cell. The increasing accuracy and availability of genomic, proteomic and systems biology approaches have allowed researchers to identify and understand how specific metabolic pathways are deregulated as a result of a disease state. The identification and modulation of key proteins, located at crucial junctions that can control metabolic flow, would offer promising therapeutic candidates for a number of disease states [1]. *Saccharomyces cerevisiae*, due to its large number of mammalian homologues and gene similarity together with ease of manipulation, has proved a model organism for basic research into the metabolic functions and regulation of particular proteins [2, 3].

One common basic characteristic to all types of cancers is reprogramming of energy metabolism to generate ATP through intense glycolytic flux with enhancement of lactate production and decreased respiration rate in spite of oxygen availability, a phenomenon known as “the Warburg effect” [3, 4]. Today we know that this metabolic adaptation is not due to mitochondrial damage since under certain circumstances cancer cells can switch back to respiration and grow faster. *S. cerevisiae* experiences the “Crabtree effect,” the ability to repress respiration and oxidative phosphorylation in response to glucose and changing to respiratory metabolism when glucose availability decreases, a behavior resembling that of cancer cells [5]. The prevailing paradigm is that cancer cells achieve a compromised balance between energy production and synthesis of macromolecules from glycolytic precursors [6] and the regulatory mechanisms behind this peculiar behavior are a hot topic in cancer research. *S. cerevisiae* could prove particularly suitable as model organism to study the regulatory key points governing this metabolic remodeling [3].

Iron serves as a cofactor for a wide variety of cellular processes, including oxygen transport, cellular respiration, the tricarboxylic acid (TCA) cycle, lipid metabolism, synthesis of metabolic intermediates, gene regulation, and DNA replication and repair [7]. Complex biosynthetic pathways are used for the assembly of Fe-porphyrin (Heme) and Fe–S clusters, essential cofactors of a large number of important enzymes. Alterations in iron homeostasis underlie many human diseases, including Friedreich's ataxia, hereditary hemochromatosis, aceruloplasminemia, Parkinson's disease, microbial pathogenesis, and cancer, as well as aging [8].

Our understanding of many of the known metabolic disorders concerning iron and its relationship to the mitochondria comes directly from yeast studies [7]. These pathways are tightly regulated due to the potential for the excessive production of reactive oxygen species (ROS) as a result of electron leakage. ROS are necessary for normal cell function and signalling, essentially by reversible redox modifications of specific cysteine residues on key proteins. This offers a quick and effective means for controlling a wide and diverse range of biological functions within the cell, whether by direct modulation of the catalytic sites, facilitating cofactor or substrate binding thus modulating their conformation or regulatory role [9]. The reversible oxidation and reduction of protein thiols by disulfide oxidoreductases with conserved active sites, such as thioredoxins (Trx's) and glutaredoxins (Grx's), can alter the functions of enzymes, receptors, transporters, and transcription factors [10]. The formation of protein mixed disulfides with glutathione (protein-SSG) is a specific form of redox modification called glutathionylation whose reversibility or deglutathionylation is primary catalyzed by Grx's [11]. Aberrant regulation of protein glutathionylation/deglutathionylation reactions due to changes in glutaredoxin activity can disrupt both apoptotic and survival signaling pathways [12].

A recent report demonstrated increases in ROS in human lung cancer cells caused the oxidation of a Cys358 of pyruvate kinase M2. As a consequence, glucose flux was diverted into the PPP to generate reducing power for antioxidant defenses [13]. Using a redox proteomic approach, we had previously identified this conserved cysteine residue in the yeast isoform of pyruvate kinase as redox sensitive in response to oxidative stress (OS) [14]. We confirmed that reversible redox modification of specific Cys residues of key glycolytic proteins allows a redirection of energy metabolites towards the PPP for NADPH production and antioxidant defense, as described earlier for glyceraldehyde-3-phosphate dehydrogenase [15–17]. In a subsequent redox shotgun proteomic screen using wild type (WT) yeast and a strain lacking the oxidoreductase glutaredoxin 2 (Grx2p), uroporphyrinogen decarboxylase (Hem12p) and transketolase (Tkl1p) were detected as containing reversibly oxidized Cys residues only in the strain lacking Grx2p, indicating they are involved in thiol disulfide exchange [18]. In the approach, proteins were tryptic digested and peptides containing reversibly oxidized Cys residues were affinity purified and analysed by MS/MS. The clear differences in the redox state of specific Cys residues of Hem12p and Tkl1p suggest that they may be direct or indirect targets of Grx2p. These proteins were selected for further analysis on the basis of their crucial roles in metabolism within the cell, as described above, and to clarify their redox properties.

**Figure 1:**
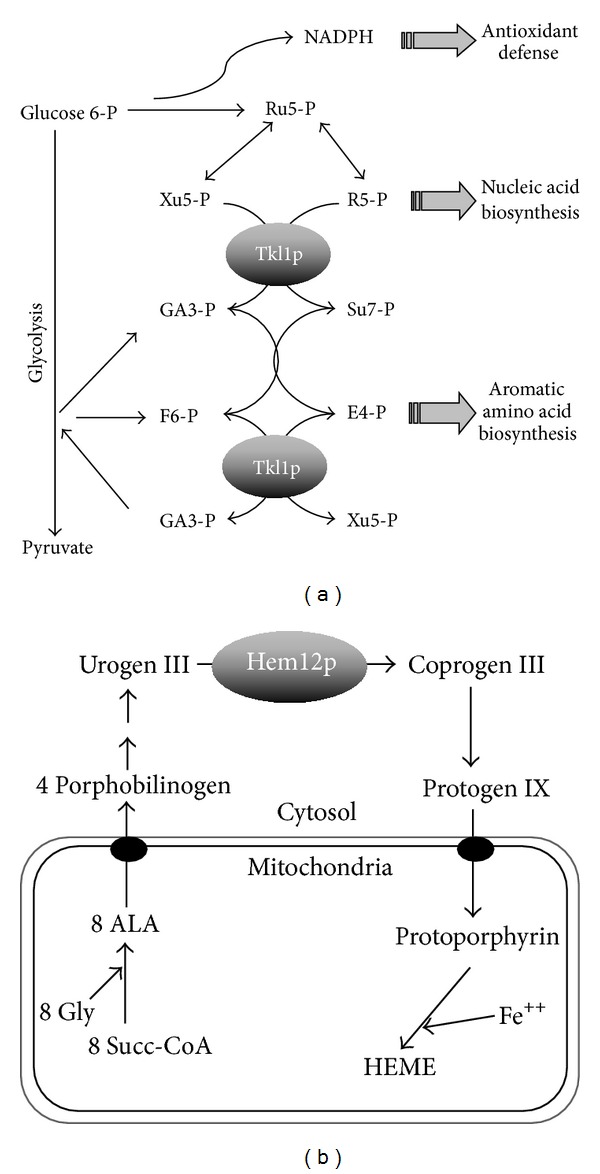
Metabolic positioning of Tkl1p and Hem12p. (a) Transketolase (Tkl1p) catalyses a number of reactions in the PPP, linking energy metabolism, antioxidant response, and biosynthesis of nucleic acids and aromatic amino acids. (b) Uroporphyrinogen decarboxylase (Hem12p) catalyses the 5th step in heme biosynthesis before biosynthesis is completed within mitochondria. Heme biosynthesis is closely linked to oxygen tension and iron homeostasis. ALA, 5-aminolevulinic acid; Urogen, Coprogen, and Protogen stand for uroporphyrinogen, coproporphyrinogen, and protoporphyrinogen, respectively. Other abbreviations are those usually employed for common metabolites.

In the nonoxidative part of the PPP, transketolase is a key enzyme that is located at the metabolic junction between the glycolytic and the PPP, which makes it an ideal candidate for the regulation of metabolic flux (Figure 1(a)). Its role is widespread across all life kingdoms as it also plays a similar role in the Calvin Cycle in plants. The PPP is essential for the production of NADPH, ribulose 5-phosphate needed for nucleotide biosynthesis, and erythrose 4-phosphate needed for aromatic amino acid biosynthesis. The reactions catalyzed by Tkl1p are essential for the diversion of the glycolytic metabolic flux toward biosynthetic pathways, which are especially active in rapidly proliferating cells. Not surprisingly, transketolase activity is increased in tumour cells [19].

Cancer therapies are increasingly being focused on the biochemistry of cancer cells as opposed to their genetic origins [20], hence understanding of regulatory mechanisms is essential to effectively target these cells [21].

Uroporphyrinogen decarboxylase, Hem12p, is involved in the 5th step of heme biosynthesis in the cytosol, before biosynthesis is completed within mitochondria (Figure 1(b)) [22]. Beside its well-known roles in oxygen transport, electron transfer, and peroxide metabolism, heme is central to oxygen sensing in many living organisms and plays a signaling role in a wide array of biological processes [23]. Altered heme expression and accumulation of heme intermediates, are responsible for a number of diseases generally referred to as porphyrias. Defects in the activity of the human orthologue of Hem12p, UroD, are associated with hepatoerythropoietic porphyria [24]. Overexpression of UroD has been recently demonstrated in tumour biopsies of patients with head and neck cancer [25].

In this study we characterized the redox properties of these enzymes from an *in vivo* experiment in *S. cerevisiae* and after exposure to H_2_O_2_. Hem12p and Tkl1p contain specific Cys residues that are redox sensitive, but yet they differ in their response to OS. To further investigate their properties* in vitro *we produced recombinant versions of these proteins and characterized their redox properties by traditional biochemical assays in combination with high-resolution proteomics to both identify and quantify the oxidative modifications on specific Cys residues.

## 2. Material and Methods

### 2.1. Materials

All reagents and chemicals were obtained from Sigma unless stated and were of analytical grade or above. The buffer used throughout unless stated was 50 mM Tris-Cl, pH 8.0. Recombinant Hem12p and Tkl1p were produced by Abyntek Biopharma S.L. (Spain). Proteins were overexpressed in *E. coli* using synthetic DNA with codon optimization, C-terminal tagged with poly-histidine, and purified by affinity capture on Ni-Sepharose.

### 2.2. Strain, Growth Conditions, and Analysis of the “Thiol Redox Peptidome”

*S. cerevisiae* strains used for redox peptidome analysis were CMML235 (WT), *MATa*, *ura*3-52, *leu2*Δ1, *his3*Δ200, and MML44 (ΔGrx2), CML235 + *grx2*::*LEU2*, a kind gift from Professor E. Herrero, University of Lleida, Spain [27]. Cells were grown on YPD medium at 30°C until exponential phase (optical density of ~1 at 600 nm) and were treated (or not) with 1 mM H_2_O_2_ for 30 min. Shaking was set at 180 rpm and 1.5 L conical flasks containing 250 mL of media were used. Cells from three independent biological replicates were harvested and the reversibly oxidized Cys containing peptides were isolated and identified as described previously [14, 18]. Briefly, in cell extracts free thiols were initially blocked with N-ethylmaleimide (NEM), and reversibly oxidized thiols were then reduced and subsequently labelled with thiol specific, biotin-HPDP. Proteins were tryptic digested and those peptides containing reversibly oxidized Cys residues were affinity purified on avidin Sepharose and analysed by MS/MS. These peptides were used to identify proteins and to map the reversibly oxidized cysteines.

### 2.3. Mass Spectrometry Analysis

Initial mass spectrometry analysis was performed as before [18]. Samples containing the affinity purified peptides were subjected to complete evaporation in a Speedvac centrifuge and then suspended in 20 *μ*L of 5% acetonitrile/2% formic acid. 5 *μ*L was used for the analysis in a Surveyor HPLC-LTQ Orbitrap XL (Thermo, USA) instrument equipped with a nanospray source. Peptides were trapped and cleaned in an Agilent 300SB-C18 5 × 0.3 mm trap column (Agilent Technologies, Germany) at a flow rate of 10 *μ*L/min of 5% acetonitrile/0.1% formic acid for 15 min and then resolved in a Biobasic-18 100 × 0.075 mm column (Thermo, USA) at a flow rate of 0.3 *μ*L/min postsplitting for 60 min using an acetonitrile gradient, supplemented with 0.1% formic acid, from 5 to 40%. The spray voltage and the capillary temperature of the MS were set to 2.0 kV and 170°C, respectively. Over the total analysis time, one Full Scan in FT mode at resolution 30000@400 m/z in the range 400–1500 m/z followed by five CID activated MS/MS in dependent mode corresponding to the five most abundant masses, was acquired. Dynamic exclusion was set to on.

For MALDI-TOF/TOF analysis peptides were desalted and concentrated by using *μ*C-18 ZipTip columns (Millipore) and directly loaded onto the MALDI plate using *α*-cyano hydroxycinnamic acid as the matrix. Mass analysis of peptides of each sample was performed with a MALDI-TOF/TOF (4800, AB Sciex) mass spectrometer using *m/z* range up to 10,000, with an accelerating voltage of 20 kV. Spectra were internally calibrated with peptides from trypsin autolysis (M + H^+^= 842.509, M + H^+^= 2211.104). Dynamic modifications included for Cys residues included NEM, glutathionylation, oxidation (–SOH), dioxidation (–SO_2_H), and trioxidation (–SO_3_H).

### 2.4. Label-Free Quantification

The relative quantification of detected Cys containing peptides was performed from affinity purified reversibly oxidized tryptic peptides from cell extracts and separately from recombinant proteins using Progenesis software (Version 3.0, Nonlinear Dynamics, UK), a label-free quantification programme for MS data [28]. The data from 3 independent replicates of the MS scans and MS/MS spectra were transformed to peak lists with Progenesis LC-MS using a proprietary algorithm and then stored in peak lists comprising *m/z* and relative abundance that show significant differences in the peak areas of parent ions using an ANOVA (*P* value < 0.05) analysis within the programme. One sample was set as a reference, and the retention times of all other samples within the experiment were aligned to this sample. In the case of the recombinant proteins after different treatments, the relative oxidation state of the Cys residues was calculated by taking the sum of the abundance of the Cys containing peptide with appropriate modification and normalising it against the abundance of the whole protein, that is, the relative abundance of all peptides detected corresponding to that protein. This is explained in more detail in Supplementary Information.

A selective reaction monitoring (SRM) experiment with appropriate proteotypic peptides for Hem12p and Tkl1p was performed to determine if there were significant differences in their concentrations between yeast strains. Initially, the recombinant proteins were diluted, tryptic digested, and analysed in a triple quadrupole mass spectrometer (4000 QTrap, ABI Sciex). The diluted proteins and their peptides were quantified using Progenesis label-free quantification (Figure S1). Five proteotypic peptides and appropriate transitions were selected from http://www.SRMatlas.org/ [29], for each recombinant protein. The major transition for each peptide was used in a SRM analysis for the relative abundance of each protein in WT and ΔGrx2 yeast strains. Two independent biological replicates of the yeast strains were analyzed by positive ion ESI LC-MS^2^ on a triple quadrupole mass spectrometer (4000 Qtrap, ABI Sciex). Samples were cleaned on a Zorbax 300SB-C18, 5 mm × 0.3 mm trap column (Agilent Technologies) for 5 min at a flow rate of 10 *μ*L/min and 95% solvent A (solvent A: 0.1% formic acid on water; solvent B: 0.1% formic acid on acetonitrile). Peptides were separated in gradient mode using a flow rate of 300 nL/min over a 75 *μ*m × 150 mm Biobasic C18 column (Thermo). Gradient was as follows: 60 min from 5% to 40% B, 10 min from 40% to 65% B. Eluted peptides were directly electrosprayed into the mass spectrometer at ESI voltage = 2800 V. The instrument was set up to cycle through all SRM transitions, followed by one enhanced resolution scan (ER) of the most prominent mass and one enhanced product ion scan (EPI, with Q0 trapping activated and scan rate of 1000 amu) of the selected mass. For SRM transitions, Q1 resolution was set to high (resolution = 2500, FWHM = 0.4 Da at *m/z* = 1000) and Q3 resolution was set to unit (resolution = 1700, FWHM = 0.6 Da at *m/z* = 1000). Dwell time for all MRM transitions was set to 50 ms. Specific instrument settings were as follows: Declustering Potential (DP) = 100, EP = 10, Curtain Gas (CUR) = 10, CAD gas = 12, Interface Heater Temperature (IHT) = 80°C. Peak detection and automatic quantification methods were built using Analyst 1.4.2 software (AB Sciex). To ensure correct peak identification and quantification, peak detection was inspected visually for coelution, similar shape, and for retention order.

### 2.5. *In Vitro* Redox Interconversion

**Figure 2:**
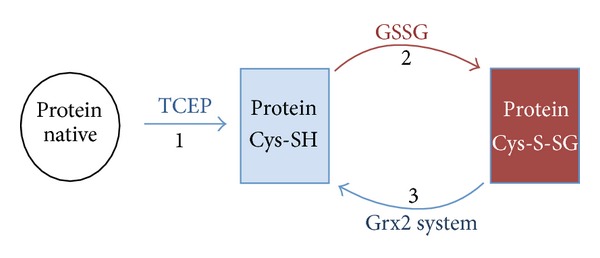
Scheme for recombinant protein redox analysis. (1) Proteins are initially reduced with TCEP and an aliquot is removed and blocked with NEM; (2) the remaining protein is glutathionylated with a high concentration of GSSG, and an aliquot is removed and any remaining free thiols are blocked with NEM; (3) the protein aliquots removed are desalted and incubated with the Grx2p system and newly formed free thiols are blocked with NEM. Aliquots prepared are used for analysis by MS/MS and free thiol determinations using DTNB.

*In vitro* redox conversion of recombinant proteins was performed as described previously [30] and outlined in Figure 2. Briefly, recombinant protein was initially reduced with 50 *μ*M TCEP (*tris*(2-carboxyethyl)phosphine) for 30 min; the mixture was incubated with 20 mM GSSG or dieosinediglutathione (DiEGSSG) or Tris buffer for controls at 37°C for 30 min. After incubation, excess GSSG was removed by Zeba spin Desalting Columns (Thermo). In the case of Tkl1p, thiamine pyrophosphate (TPP) and MgCl_2_ were maintained in the buffer and subsequently added to the mixture after any desalting procedures. For deglutathionylation, following treatment with GSSG, the proteins were incubated with Grx2p mix containing, 5 nM yeast recombinant Grx2p [31], 0.4 mM NADPH, 0.75 mM GSH, and 80 nM glutathione reductase (GR) (all Sigma). Remaining reduced thiol groups were subsequently blocked with NEM at all stages. Aliquots of the mixture containing 10 *μ*M of protein were subjected to 12% SDS-PAGE electrophoresis under nonreducing conditions. Gels were stained with Coomassie R-250 and duplicate gels were transferred to nitrocellulose membranes and reversibly stained with Ponceau S to ensure complete transfer. Membranes were blocked with 2% BSA in tris buffered saline containing 0.5% tween (TBS-T) and incubated with primary antibody anti-GSH (Virogen, Watertown, USA) using a dilution of 1 : 1500. Membranes were washed with TBST and incubated with secondary goat anti-mouse (Sigma) at a concentration of 1 : 4000 and the chemiluminescent signal was detected by using a LAS-3000 camera (Fujifilm, Tokyo).

 Free thiol content of proteins was estimated using Ellman's reagent (5,5-dithiobis-2-nitrobenzoic acid, DTNB) at 412 nm in Tris buffer pH 8.0 [32]; measurements were performed from three independent preparations. MS/MS analysis of recombinant proteins was performed as described with separate aliquots of the protein mixtures digested with sequencing grade trypsin (Promega) and stored at −70°C.

### 2.6. Fluorescent Deglutathionylation

DiEGSSG was from IMCO Ltd (Stockholm, Sweden) and a kind gift from Professor Arne Holmgren, Karolinska Institute, Sweden. This glutathione disulfide derivative has low fluorescence due to self-quenching, while the reduced eosin-glutathione (eosin-GSH) has high fluorescence. Protein concentrations of recombinant proteins were determined using absorbance at 280 nm and the appropriate molar extinction coefficient for each protein (BSA = 43824 M^−1^ cm^−1^, Hem12p = 58900 M^−1^ cm^−1^, and Tkl1p = 88240 M^−1^ cm^−1^). Proteins were incubated with excess DiEGSSG and labelling with eosin-GSH was monitored using the molar extinction coefficient of eosin isothiocyanate (EITC) at 525 nm (=56000 M^−1^ cm^−1^). 1 *μ*M of fluorescently labelled protein was incubated with “Grx2p mix” as above for over 5 min. Kinetic measurements were recorded over 5 min in a Synergy HT (Bio-Tek) 96-well fluorescence plate reader with excitation at 485 nm and emission 528 nm. Positive control used was BSA labelled with eosin-GSH with complete Grx2p system (IMCO, Sweden) and negative control was a duplicate but lacking Grx2p system. Deglutathionylation activity by Grx2p was determined from three independent redox interconversion preparations for each recombinant protein and also measured in duplicate.

### 2.7. Transketolase Activity

Activity was determined by oxidation of NADH at 340 nm over 5 min in a coupled reaction as described previously [33]. Briefly the reaction mixture containing xylulose 5-phosphate (2 mM), TPP (10 *μ*M), MgCl_2_ (1.2 mM), triosephosphate isomerase (1 unit), glyceraldehyde phosphate dehydrogenase (1 unit), NADH (10 *μ*M), and ribose 5-phosphate (10 mM) was allowed to equilibrate before the addition of 1.5 *μ*M of transketolase treated with or without different sulfhydryl reagents. When Tkl1p ± GSSG treated with 1 mM NEM was analysed, the reagent was carried over into the assay mixture at a final concentration of 0.01 mM, but this concentration of NEM had minimal effect on the activity of the enzyme. Duplicate preparations were performed for Tkl1p and Tkl1p + GSSG, activity was measured in triplicate, and statistics were performed using two tailed paired Student's *t*-test.

## 3. Results

### 3.1. Redox Analysis of the Proteome: Relative Quantification of the Reversibly Oxidized Cys Residues from Protein Extracts of Cell Cultures Reveals that Hem12p and Tkl1p Are Redox Sensitive Proteins

Reversibly oxidized Cys containing tryptic peptides of cell extracts from both WT and ΔGrx2 and after exposure to 1 mM H_2_O_2_ for 30 min were affinity purified, analysed, identified by MS/MS, and relatively quantified using the label-free quantification programme Progenesis LC-MS (Table 1). In the case of Hem12p, only Cys26 was detected as reversibly oxidized in cell extracts and there was no significant change in the signal intensity of the parent ion of the peptide containing Cys26 after H_2_O_2_ treatment, yet there was a sixfold increase in the amount of reversibly oxidized Cys26 parent ion in the ΔGrx2 strain.

In the case of Tkl1p only one of the two Cys residues, Cys622, could be detected. The tryptic peptide containing reversibly oxidized Cys622 was detected with a higher abundance in the ΔGrx2 strain but more significantly in both strains after H_2_O_2_ treatment. We relatively quantified the proteins between strains using SRM to confirm that the increase in the detection of the Cys redox peptides was due to oxidation of the Cys residue and not to an increase in the protein concentrations.  Table 2 lists the proteotypic peptides, the appropriate transitions used for each peptide, and the ratio of detection between strains. Despite some variability on the behaviour of the proteotypic peptides and transitions selected, it is clear that a change in protein total concentration cannot account for the significant increase in abundance of the oxidized Cys peptides detected in the ΔGrx2 strain compared to WT. According to these results, both Hem12p and Tkl1p showed a true redox response on their Cys residues upon H_2_O_2_ treatment and/or Grx2p depletion.

A striking increase in the oxidized form of Cys-peptides was the common response of both enzymes to the absence of Grx2p, suggesting they could be prone to glutathionylation [11]. To check this possibility, we prepared the recombinant forms of Hem12p and Tkl1p and studied the glutathione dependent redox interconversion *in vitro* by both redox proteomic and biochemical strategies. A scheme for the strategy employed is outlined in Figure 2. Proteins were initially reduced using a trace of the thiol reductor TCEP and glutathionylated using a high concentration of oxidized glutathione (GSSG), a strategy widely employed by many groups [34, 35]. The proteins were desalted to remove excess GSSG and subsequently incubated with the Grx2p system (Grx2p, glutathione reductase (GR), NADPH, GSH) to monitor deglutathionylation. Aliquots are taken at each stage with remaining free thiols blocked with NEM before analysis by MS/MS.

### 3.2. Hem12p: Cys52 of Hem12p Can Be Glutathionylated and Subsequently Deglutathionylated by Grx2p *In Vitro*

**Figure 3:**
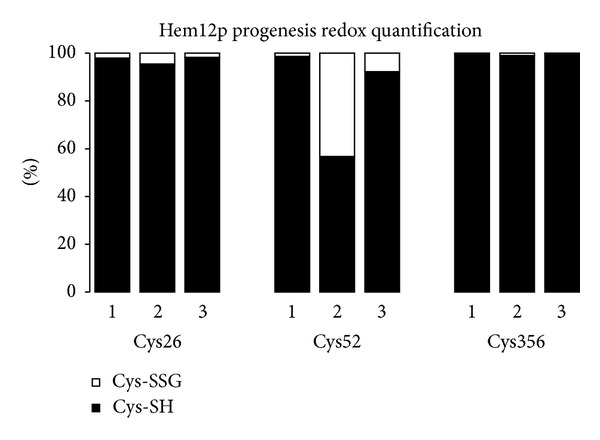
Redox interconversion of Hem12p. Hem12p was initially reduced with TCEP followed by either incubation with NEM (sample 1), incubation with GSSG followed by NEM (sample 2), incubation with GSSG followed by Grx2p system, and then NEM (sample 3). Progenesis quantification of modified thiol groups was carried out using the signal intensity of parent ions for the treated recombinant protein. Recombinant protein quantification for each treatment is normalized to all peptides detected and quantified for the recombinant protein (see Supplementary Information available online at http://dx.doi.org/10.1155/2013/932472 for further description of the method).

**Figure 4:**
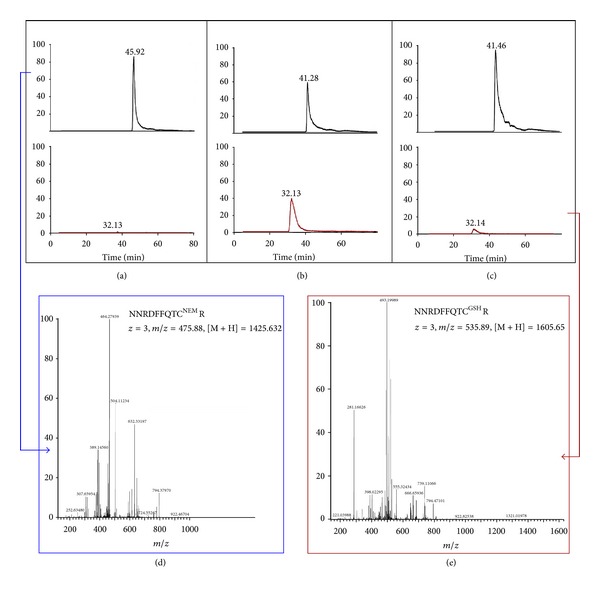
MS analysis of recombinant Hem12p. (a) Representative extracted ion chromatograms (XIC) of peptide NNRDFFQTCR with Cys52 of Hem12p (a) reduced, (b) +GSSG, (c) +Grx2p system. Traces in the upper panels correspond to the reduced peptide followed by alkylation with NEM (+125); traces in red in the lower panel correspond to the peptide glutathionylated (+305). (d) Fragmentation spectra of the parent ion in reduced state (+NEM) and (e) with GSH are presented.

**Figure 5:**
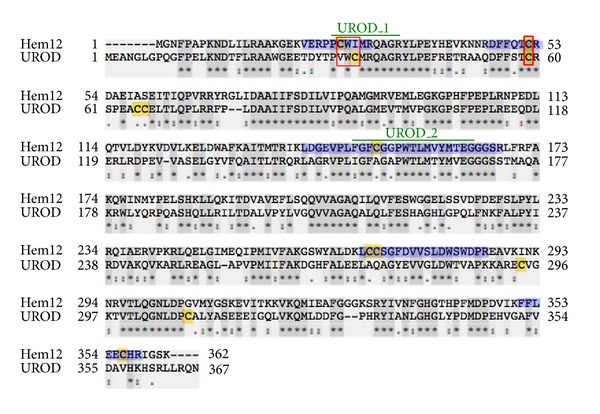
Alignment of uroporphyrinogen decarboxylase from *S. cerevisiae* and human. Hem12p is the enzyme from *S. cerevisiae *UniProt P32347 and UROD is from human, UniProt P06132. Cysteines are highlighted in yellow; dark grey shadowed residues indicate identical positions; tryptic Cys-peptides in Hem12p are highlighted in blue; thin red square marks Cys26 and its equivalent Cys35 in UROD; thick red square marks the conserved cysteine, Cys52 in Hem12p and Cys59 in UROD, respectively; green lines indicate the sequence signatures for Uroporphyrinogen decarboxylase UROD_1 and UROD_2 (PS00906 and PS00907, resp., according to ProSite). The sequences have been aligned using the program Clustal-*ω* and share 49.60% identity.

Treatment of Hem12p with GSSG produced a glutathionylated version of the protein as detected initially in a preliminary analysis by Immunoblotting (not shown). Following the scheme outlined in Figure 2, we obtained 3 samples of the protein: (1) fully reduced, (2) GSSG treated (glutathionylated), and (3) incubated with a Grx2p system (theoretically deglutathionylated). Tryptic digestion of these samples, analysis by MS/MS, and quantification of the total protein and individual peptides containing Cys confirmed the identification of the glutathionylated Cys residues (Figures 3 and 4). In this analysis, the most susceptible identifiable residue to glutathionylation is Cys52 and incubation with the Grx2p system resulted in almost complete deglutathionylation of the residue. Hem12p contains 6 Cys residues within 5 tryptic peptides, where 1 peptide contains 2 Cys residues (Figure 5). We detected 4 of the 5 peptides, the remaining peptide containing 2 Cys residues being nonamenable to MS/MS detection due to size or amino acid composition (peptide containing Cys126 did not fragment well and was not included in analysis). Reduced Hem12p had an average free thiol content of 4.5 *μ*M/*μ*M protein and Hem12p treated with GSSG had an average free thiol content of 1.25 *μ*M/*μ*M protein as determined by titration with DTNB, indicating that *≈*3 Cys residues were oxidized per protein molecule. Therefore, we also cannot rule out (de)glutathionylation of Cys270/271 or Cys126.

Inspection of the extracted ion chromatograms for the Cys52 peptide confirmed that the parent ion containing the glutathione moiety is almost completely reduced after incubation with Grx2p system (Figure 4).

**Figure 6:**
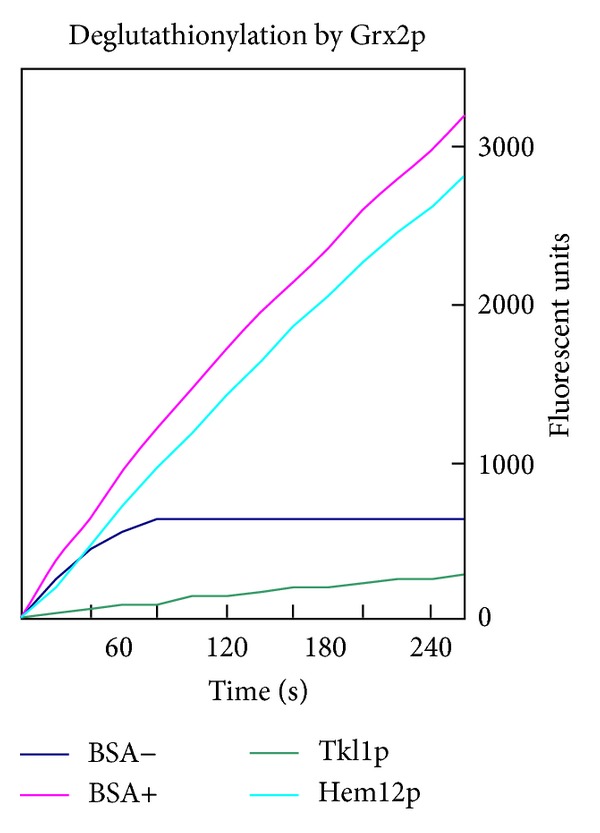
Deglutathionylation of fluorescently labelled proteins (with eosin-GSH) by Grx2p system. Increase in fluorescence emission by free eosin-GSH is given in arbitrary units. BSA labelled with eosin-GSH and then incubated with complete Grx2p system or not are used as the positive (BSA+) and negative (BSA−) controls, respectively.

In a separate assay to confirm deglutathionylation by Grx2p, we used a fluorescent GSSG moiety (DiEGSSG), where fluorescence is quenched when it is part of a disulfide and fluorescence is liberated in the form of eosin-GSH [36]. We labelled Hem12p with this reagent and calculated, using appropriate molar extinction coefficients, that for every mole of Hem12p, there was ~1.5 moles of fluorescent glutathione attached. We monitored the activity of the deglutathionylation of Hem12p in the presence of the Grx2p system by the increase in fluorescence, showing the same rate as the reference protein, glutathionylated BSA (Figure 6). These results demonstrate that glutathionylated Cys52 in uroporphyrinogen decarboxylase is a substrate for Grx2p.

 Unfortunately, we were unable to source or produce the reagents necessary to measure the activity of this enzyme, but from previous studies it was shown that the activity of Hem12p from *S. cerevisiae* is at a maximum in the presence of reducing agents such as dithiothreitol, and inhibited by sulfhydryl agents [37]. Together these results would suggest that the glutathionylation and subsequent reversible redox dependent control would provide an excellent means for the cell to protect this constitutively expressed enzyme and provide a mechanism for the control of heme biosynthesis dependent on the prevailing redox environment.

### 3.3. Tkl1p: Reversible Oxidation of Cys622 of Tkl1p Is an Oxidative Stress Response

Tkl1p contains two Cys residues. Cys159 is part of the active site with a role in TPP binding and highly conserved across different species [38]. As shown in Table 1, the relative abundance of the peptide containing reversibly oxidized Cys622 was highest in H_2_O_2_ treated cells, with the ΔGrx2 strain having the highest signal intensity. 

**Figure 7:**
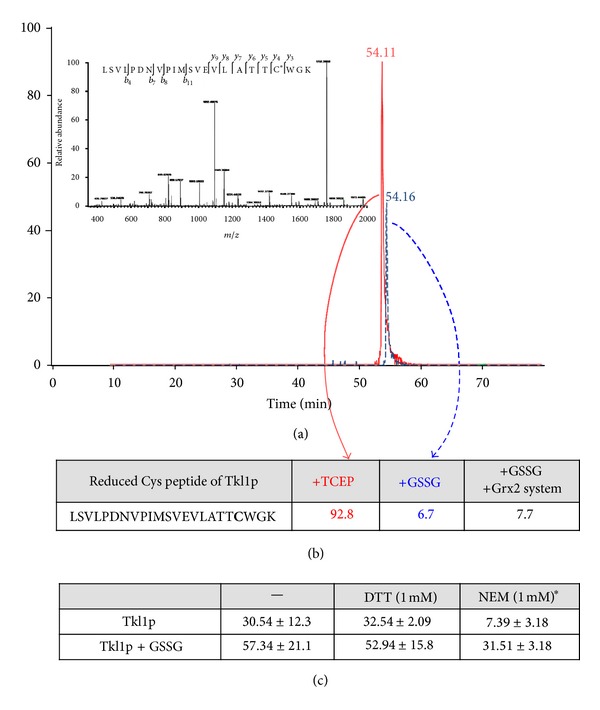
Redox interconversion and activity of Tkl1p. (a) Representative extracted ion chromatogram and fragmentation (inset) of tryptic peptide from Tkl1p containing Cys622 (reduced) after treatment with TCEP (red) or GSSG (blue), followed by NEM. (b) Progenesis quantification of reduced peptide after treatments in the presence of Grx2p system (values in arbitrary units × 10^6^). (c) Transketolase activity after redox treatments, *≈*80% inhibition of enzyme activity after pretreatment with NEM, significance *P* < 0.05. *The final concentration of NEM in the assay was 0.01 mM; this concentration had little effect on activity. Units are *μ*mol min^−1^/*μ*g protein.

When recombinant Tkl1p was analysed it was not detected as glutathionylated in a preliminary analysis by western blotting following the same scheme outlined in Figure 2 (not shown). The tryptic peptide containing Cys159 is 77 amino acids long and we were unable to detect this peptide, either by electron spray MS/MS or MALDI-TOF/TOF. We also attempted to detect this Cys residue using alternative proteases and again were unable to detect a peptide containing Cys159. Cys622-containing peptide was only detected in its reduced state, with a tenfold lower intensity of the reduced peptide after GSSG treatment than when it was treated with TCEP. Extracted ion chromatograms of the reduced peptide are presented in Figure 7(a) and their quantitative analysis in Figure 7(b). The decrease in the amount of reduced peptide is quantitatively comparable to the increase in the oxidized peptide in extracts of cell cultures after H_2_O_2_ exposure (see Table 1). However, the oxidized form of the peptide could not be identified at this point.

**Figure 8:**
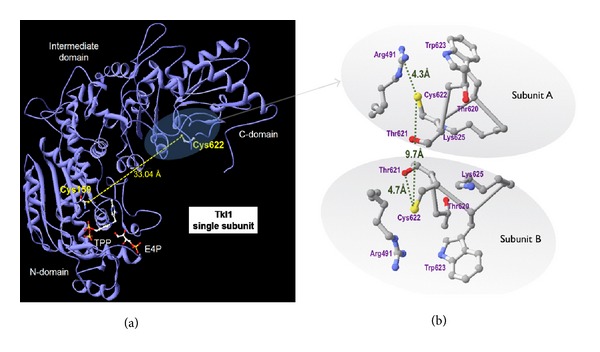
Positioning of Cys622 in the structure of the Tkl1p molecule. Structure of Tkl1p with bound TPP, E4P, and Ca^2+^. (a) Subunit of homodimer, distance between cys622 and cys159 of active site from either subunit is too large for intradisulfide. (b) Interphase between subunits of homodimer, distance between cys622 of homodimers is 9.68 Å; Cys622 is close to both Thr and Arg residues, which can favour a thiolate and formation of sulfenic acid. Figures were prepared from structure PDB 1NGS using the free software DeepView [26].

The published structure of *S. cerevisiae* Tkl1p (PDB: 1GNS) with TPP cofactor, E4-P and Ca^2+^, indicates it is a homodimer and that the Cys622 detected by our redox proteomic approach is accessible in the dimer (Figure 8(a)). Incubation of the enzyme with TPP is necessary for correct folding and activity, with the number of accessible free thiol groups dependent on TPP binding [39]. Intersubunit contacts are confined to the N-terminal and intermediate domains, whereas C-terminal domains have few contacts and their packing is very loose with a large “tunnel” at the interface. Moreover, the C-terminal domain does not contribute to TPP and substrate binding, but a possible regulatory role for this domain was proposed [40]. Cys622 of both subunits is in a loop at the interface, with their –SH side chains pointing away from each other and separated by *≈*9 Å [41]. In the crystal structure, the Cys622 residue is surrounded by positively charged amino acids. According to the structure of the dimer, the distance between the nonconserved Cys622 and the active site is >30 Å, which would normally prohibit a direct influence on the catalytic mechanism. Cys622 is ~4 Å from Arg491 (Figure 8(b)), which could stabilize a possible thiolate at Cys622 and is preceded by two Thr residues (Thr620 and Thr621), favourable to the formation of a stable sulfenic acid on Cys622 [42].

Using electron spray ionisation and MS/MS we were unable to detect the Cys622 tryptic peptide with glutathione or as a sulfenic acid (or further oxidation states). However, analysis by MALDI-TOF/TOF allowed the detection of the peptide containing Cys622 in the sulfenic acid form (+16 Da), and with a mass difference of +305 Da, corresponding to glutathionylation after GSSG treatment (Figure S2).

Comparison of the enzymatic activity of reduced Tkl1p and treated with GSSG surprisingly resulted in higher activity of the enzyme after GSSG treatment (Figure 7(c)). Treatment of the reduced Tkl1p with NEM resulted in a significantly lower activity of the enzyme indicating that free thiols are needed for activity. However, pretreatment of the enzyme with GSSG protected against almost complete inactivation as compared to NEM alone. Preincubation of the enzyme with DTT did not significantly alter the activity of the enzyme and had little effect on the activity of Tkl1p treated with GSSG (Figure 7(c)). Consistent with this result, estimation of the free thiol content of the reduced protein and of that subsequently treated with GSSG failed to show any significant differences (not shown). Moreover, incubation of the enzyme with DiEGSSG resulted in partial glutathionylation with ~0.35 moles of the fluorescent moiety for every mole of Tkl1p. As to be expected the fluorescent deglutathionylation of the treated enzyme by the Grx2p system as measured by an increase in fluorescence was ~10-fold lower than for Hem12p or the positive control BSA (Figure 6).

Together these results suggest that treatment of the enzyme with GSSG results in a conformational change accompanied by limited glutathionylation of Cys622 and marked enhancement of the enzymatic activity. This indicates that glutathionylation is probably not a natural redox modification of this enzyme. However, the plant orthologue of this enzyme has previously been identified as a Trx target [43] and our results would suggest that the protein is involved in a thiol disulfide exchange mechanism.

## 4. Discussion

Cys residues due to their various oxidation states can influence the activity of enzymes, whether they form part of the catalytic site, involved in cofactor binding or allosteric modulation [9, 44]. Proteomics has developed into a powerful tool not only for the detection and identification of proteins, but recent advances have allowed the identification and quantification of posttranslational modifications on specific residues of redox proteins [45]. In a previous shotgun proteomic screen to detect potential Grx2p protein targets two proteins, Hem12p and Tkl1p, were identified as containing reversibly oxidized Cys residues in a ΔGrx2 strain but not in the corresponding WT [18]. Both proteins function at critical junctions in iron regulation and biosynthetic metabolism, respectively. In this study we applied proteomic approaches to characterise the redox properties of these proteins and confirm targets of thiol disulfide exchange with Grx2p.

Cys26 of Hem12p is located inside a characteristic uroporphyrinogen decarboxylase sequence signature, UROD_1, which, according to the literature and modelling, is placed near the catalytic site and involved in substrate binding [46] (see Figure 5). In the ΔGrx2 yeast strain there was a 6-fold increase in the abundance of reversibly oxidized Cys26, yet SRM showed that overall protein abundance did not change significantly between strains, indicating the reversible oxidation was a true redox posttranslational modification in response to the lack of Grx2p. However, this cysteine residue was not sensitive to GSSG treatment of the enzyme *in vitro*, suggesting that the redox change observed *in vivo* could follow a different mechanism. Whether another thiol oxidoreductase is involved would be an interesting matter for further research.

Hem12p-Cys52 is involved in substrate binding and is highly conserved across species but is not essential for catalytic activity [28]. In the recombinant protein, Cys52 could be glutathionylated and deglutathionylated *in vitro*, indicating it is a model substrate for deglutathionylation by Grx2p. These results further strengthen the links between glutaredoxins and the tight control of iron homeostasis within the cell. Hem12p is a constitutive protein that catalyses the 5th step in heme biosynthesis in the cytosol before synthesis is completed within the mitochondria. Heme biosynthesis needs to be tightly regulated within the cell to prevent overproduction of toxic porphyrins and is correlated to oxygen tension within the yeast cell [23]. The biosynthesis of Fe/S clusters is also tightly coordinated with that of heme, and in yeast deletion of genes important for Fe/S assembly negatively affects heme synthesis [47]. The glutaredoxin family of proteins has been clearly implicated in iron control with the monothiols Grx3p, Grx4p, and Grx5p playing key roles in iron regulation [27, 48, 49]. We previously detected changes in iron homeostasis in strains lacking Grx2p [18]. The potential covalent regulation of the activity of the constitutive enzyme Hem12p by thiol disulfide exchange would provide a rapid means to control heme biosynthesis as a function of cytosolic or mitochondrial redox state as compared to a slower control mechanism carried out by transcriptional regulation at other points in the pathway. 

Cys622 of Tkl1p is highly sensitive to redox changes; its oxidized form increases > 4-fold induced by the lack of Grx2p and > 6-fold by H_2_O_2_ *in vivo*, without any increase in the total amount of the protein. Only a relatively low proportion of the recombinant protein could be glutathionylated *in vitro* and we could only detect one of the two Cys containing tryptic peptides within the protein. Interestingly, preincubation of the protein with GSSG affects the activity of the enzyme and protects deactivation by the sulfhydryl reagent NEM, indicating a thiol protective effect. These results support a thiol disulfide exchange mechanism controlling the activity of the protein, but our results suggest that it is not a natural direct target of Grx2p. Transketolase is an ideal node for redox control within the cell. Its activation under oxidative conditions would be coherent with diversion of metabolic flux toward the PPP to provide reducing power for antioxidant defense systems. Moreover, the products of transketolase reaction form starting points for several other biosynthetic pathways, thus redistributing the fate of glucose derived metabolites towards macromolecular building blocks for cellular proliferation (see Figure 1(a)).

Using a redox proteomics approach we have shown that Cys296 of yeast pyruvate kinase, located at the allosteric activator domain, was sensitive to oxidative conditions and we suggested a role in adaptation to oxidative stress [14]. Similar behaviour of the equivalent Cys358 in human pyruvate kinase PKM2 was subsequently demonstrated to be the basis of a redox regulatory mechanism for metabolic remodelling that allowed cancer cells to resist oxidative stress and proliferate [13]. In the same way, the redox sensitivity of human uroporphyrinogen decarboxylase and transketolase may be worthy of study. The two cysteines of Hem12p identified here as being redox sensitive, Cys26 and Cys52, have their counterpart residues Cys35 and Cys59 in UROD, the human orthologue (see Figure 5). Similarly, the human transketolase is rich in cysteine residues some of which may be prone to reversible oxidation with influence on enzyme functionality. Incidentally, overexpression of the human orthologue UROD has been associated with diseases involving porphyrin metabolism and cancer [25] and enhancement in the activity and concentration of TKLT1 are highly correlated with rate of tumour growth in a variety of cancers [50].

The redox properties of yeast Hem12p and Tkl1p described here could have an impact on the knowledge of redox regulatory mechanisms in all life kingdoms. For instance, the plant orthologues of these two enzymes have been identified as Trx targets in chloroplasts and *Chlamydomonas* [43]. Regulation of plant enzymes by thiol-disulfide exchange mediated by thioredoxin pioneered the work in the field [51]. Recently, glutathionylation and nitrosylation have also been demonstrated to operate in plants, with “redoxins” performing a crucial role [52]. In the case of uroporphyrinogen decarboxylase, redox regulation would have an influence on the biosynthetic pathways for siroheme and chlorophyll where the later has to be regulated by light/darkness fluctuations through the redox state of the chloroplast [53].

## 5. Conclusions

The results presented herein demonstrate that Tkl1p and Hem12p from yeast are sensitive to changes in the cellular redox homeostasis and uncover striking redox properties of these important enzymes involved in iron homeostasis and antioxidant and biosynthetic metabolism. These findings could reach beyond yeast to enlighten metabolic remodelling mechanisms involving their human counterparts. The door is now open to further work in this direction to describe the molecular, physiological, and pathological insights in depth.

## Supplementary Material

Supplementary Information contains a detailed description of the quantitative proteomics methodology used for Cys-containing peptides and for SRM analysis of Hem12p and Tkl1p.

## Figures and Tables

**Table 1:** Label-free MS quantification of reversibly oxidised Cys residues of Hem12p and Tkl1p using Progenesis. Peptides detected in a shotgun proteomic screen of reversibly oxidised Cys residues using the redox affinity enrichment approach in WT and ΔGrx2 yeast strains ±1 mM H_2_O_2_. Units are arbitrary for intensity of parent ions.

Protein and Cys peptide	Peak intensity of oxidized Cys peptide (arbitrary units)			
	WT	WT + H_2_O_2_	ΔGrx2	ΔGrx2 + H_2_O_2_
Tkl1p (Cys622)				
LSVLPDNVPIMSVEVLATT**C**WGK	2.3	22.9**	9.5	58.5**
Hem12p (Cys26)				
VERPP**C**WIMR	5.0	7.0	30.0*	23.2

*Hem12p had a sixfold increase in the detection of reversibly oxidised Cys26 in the ΔGrx2 strain compared to WT (*P* = 0.0042). **Tkl1p was increased in both strains after H_2_O_2_ treatment and more significantly in the ΔGrx2 strain; this peptide was detected with *z* = 2 (*P* = 0.00471) and *z* = 3 (*P* = 0.0109). Peak intensities are given in arbitrary units ×10^4^ (Tkl1p) and ×10^3^ (Hem12p).

**Table 2:** SRM quantification of Hem12p and Tkl1p in WT and ΔGrx2 yeast strains. Proteotypic peptides and transitions used for SRM analysis and relative quantification are shown. Peak intensity is given in arbitrary units ×10^4^. SRM was performed with two independent biological replicates.

Proteotypic peptide	Q1 (*m*/*z*)	Q3 (*m*/*z*)	Fragmentation	Peak intensity	
				WT	ΔGrx2
Uroporphyrinogen decarboxylase					
VTLQGNLDPGVMYGSK	839.93	838.41	2+/y8	2.35	1.66
DAEIASEITIQPVR	771.41	1113.63	2+/y10	1.87	ND
YIVNFGHGTHPFMDPDVIK	1094.04	1061.53	2+/y9	3.32	ND
NPEDLQTVLDYK	717.86	979.55	2+/y8	5.16	6.33
QMIEAFGGGK	519.26	665.33	2+/y7	2.42	ND
Transketolase					
FFGFTPEGVAER	678.83	757.39	2+/y7	9.70	4.10
QNLPQLEGSSIESASK	844.43	1332.66	2+/y13	0.63	0.18
SFVVPQEVYDHYQK	580.29	653.81	2+/y10	9.41	6.63
ANSGHPGAPLGMAPAAHVLWSQMR	819.41	919.49	2+/y7	0.22	0.23
SLPNIQVWRPADGNEVSAAYK	772.40	938.46	2+/y9	0.28	0.21

ND not detected.
