# Supplementary material for: Thiol Redox Sensitivity of Two Key Enzymes of Heme Biosynthesis and Pentose Phosphate Pathways: Uroporphyrinogen Decarboxylase and Transketolase
Source: Oxid Med Cell Longev. 2013 Jul 16;2013:932472. doi: 10.1155/2013/932472 (PMC3730168; doi:10.1155/2013/932472)
Supplement: Supplementary file 1 — Supplementary Information contains a detailed description of the quantitative proteomics methodology used for Cys-containing peptides and for SRM analysis of Hem12p and Tkl1p. [file 932472.f1.docx]

**Composition Comments**

1. Please provide the name(s) of city (ies) and valid postal code(s) to the address (es) lacking ones as per journal style.

Please edit the authors and their affiliations as follow:

**Babak Vahdatpour,1 Farshid Alizadeh,2 Amir Moayednia,1 Masoud Emadi,1 Mohammad**

**Hatef Khorami,2 and Saeid Haghdani3**

*^1^Department of Physical Medicine and Rehabilitation, Isfahan University of Medical Sciences, Isfahan, Iran*

*^2^Department of Urology, Isfahan Urology and Kidney Transplantation Research Center, Isfahan University of Medical Sciences, Unit 10, No. 22, 16th Alley, Shams Abadi Street, postal code: 81347-44134, Isfahan, Iran.*

*^3^Department of Urology, Hasheminejad Kidney Center (HKC), Tehran University of Medical Sciences (TUMS), Tehran, Iran*

*Correspondence email:* [*f_alizadeh@med.mui.ac.ir*](mailto:f_alizadeh@med.mui.ac.ir)*.*

*Tel +989133179509. Fax: +983112350532*

2. We added the highlighted part(s) to the provided address(es). Please check.

We checked, it is ok

3. Please provide a shorter abstract that does not exceed 200 words. It should be citation-free and self-contained and should include a brief summary of what is being presented in the paper, without detailed introduction of notations.

The following abstract is presented with 208 words. We think it is the briefest one.

**Abstract**

**Objectives:** To investigated the effectiveness of extracorporeal shock wave therapy (ESWT) for symptoms alleviation in chronic pelvic pain syndrome (CPPS).

**Materials and methods:** 40 patients with CPPS were randomly allocated into either the treatment or sham group. In the first group, patients were treated by ESWT once a week for 4 weeks by a defined protocol. In the sham group, the same protocol was applied but with the probe being turned off. The follow up assessments were done at 1, 2, 3, and 12 weeks by Visual Analogue Scale (VAS) for pain and NIH-developed Chronic Prostatitis Symptom Index (NIH-CPSI).

**Results:** Pain domain score at follow up points in both treatment and sham groups were reduced, more so in the treatment group which were significant at week 2, 3, and 12. Urinary scores became significantly different at weeks 3 and 12. Also, quality of life (QOL) and total NIH-CPSI scores at all four follow up time points reduced more significantly in the treatment group as compared to the sham group. Noticeably, at week 12 a slight deterioration in all variables was observed compared to the first 3 weeks of the treatment period.

**Conclusions:** our findings confirmed ESWT therapy as a safe and effective method in CPPS in short term.

4. We reformatted Table 1. Please check.

We checked, it is ok

5. Should we distinguish the values in red color in Table 1 by changing its font? Please check.

No, the presented format is ok.

6. We made the highlighted change(s) for the sake of clarity and correctness. Please check.

We checked, it is ok

7. We made the highlighted changes according to the list of references. Please check similar cases throughout.

We checked, it is ok

8. Please change mJouls/M^2^ to mJouls/mm^2^ in the Method of Treatment, page 2, the right column, line 1, and in the Discussion part, page 4, the right column, line1.
