# Supplementary material for: Thiol Redox Sensitivity of Two Key Enzymes of Heme Biosynthesis and Pentose Phosphate Pathways: Uroporphyrinogen Decarboxylase and Transketolase
Source: Oxid Med Cell Longev. 2013 Jul 16;2013:932472. doi: 10.1155/2013/932472 (PMC3730168; doi:10.1155/2013/932472)
Supplement: Supplementary file 2 [file 932472.f2.pdf]

OXIDATIVE MEDICINE AND CELLULAR LONGEVITY
932472.v2 (Research Article)
McDonagh et al.

Supplementary Information

Hem12p and Tkl1p were identified from an initial redox shotgun proteomic approach where only peptides containing Cys residues that are reversibly oxidized are selected. In this approach proteins were tryptic digested and peptides containing reversibly oxidized Cys residues were affinity purified and analyzed by MS/MS. A subset of proteins was only detected in the strain lacking Grx2p and not in its corresponding WT, as containing reversibly oxidised Cys residues. There were 3 independent biological replicates for both wild type and ΔGrx2 strains [1]. In this article we state a protein has to be detected at least twice from two independent replicates for inclusion in a list of identified proteins. The criteria for inclusion of identified peptides and to obtain a FDR of 0.048 with the decoy database was peptide score vs. charge state, charge 2, XCorr > 2.095, charge 3, XCorr > 2.52, charge 4, XCorr > 2.79. Using these criteria, oxidized Cys containing peptides from Hem12p and Tkl1p were only detected in the strain lacking Grx2p and not in the WT. To further investigate and as a complimentary approach we employed the label free quantification programme Progenesis to relatively quantify the abundance of these cys containing peptides between the two strains and after peroxide exposure.

The shotgun proteomic experiment MS/MS analysis was performed using data dependent acquisition (DDA), such that in each precursor scan the 5 most intense ions are fragmented, these are then excluded and the next 5 most intense ions are fragmented and so on throughout the dwelling time. Therefore we identified Hem12p and Tkl1p from their Cys containing peptides, however no statistics were involved here as either the peptides were present or not. Statistics were used in the Progenesis LC-MS analysis. This program quantifies at the MS level stage, not MS/MS. As not every ion is fragmented in DDA and the MS/MS spectra for parent ions that are not confidently identified are not included, this information is potentially lost. Progenesis aligns ions with the same retention time and m/z value from .raw files from all biological replicates to a reference sample. Files are then

merged into a single .mgf file, which is then used to identify only those proteins that show significant differences in the peak areas of parent ions. Therefore as with any of these methods it is necessary to select one sample as an alignment reference. All other samples then have their retention times aligned to the reference, allowing a correction for any drift in retention time. This results in adjusted retention times, which are equivalent across all runs and creates a single set of peptide ions shared by all runs. Alignment is driven by the placement of landmarks and connects the location of a particular peptide ion on the reference run with the same ions on the runs being aligned. Statistics for fold changes are calculated by an Anova analysis incorporated within the Progenesis programme, the corresponding p-values for each fold change of peptides have been included.

In the recombinant proteins the relative quantification of the oxidation state of the Cys residues after redox interconversion was calculated in a similar manner. In a traditional proteomic experiment, Progenesis sums the individual intensities of the peptides identified of a particular protein to give a value for the relative abundance of that protein. We analyzed the tryptic peptides of recombinant proteins after various redox interconversions and used the overall abundance value of the particular protein identified by all peptides, to normalize across different runs. The ion intensities of individual Cys containing peptides were summed when containing the same modification and again normalized against the total abundance of the protein to compare the relative abundance across different replicates. It must be pointed out at this stage that for any given peptide, distinct modifications such as NEM or glutathionylation may affect the ionisation of the peptide and hence their detection and quantification. In this respect, as we are dealing with recombinant proteins we assume that the average abundance of identical peptides with either modification is proportional to their concentration.

In order to select proteotypic peptides that could be detected for recombinant proteins, the recombinant proteins were diluted, digested and analyzed in the 4000 QTrap (ABI Scix). The diluted proteins and their peptides were then subjected to Progenesis label free quantification Fig. S1. Five proteotypic peptides and their appropriate major transition were obtained from www.SRMatlas.org [2] for each respective recombinant protein. The subsequent major transitions for each peptide according to www.SRMatlas.org were used

in a SRM analysis for the relative abundance of each protein in a wild type and ΔGrx2p yeast strains. This was performed from two independent biological replicates of each strain.

[1] B. McDonagh, C.A. Padilla, J.R. Pedrajas, J.A. Bárcena, Biosynthetic and iron metabolism is regulated by thiol proteome changes dependent on glutaredoxin-2 and mitochondrial peroxiredoxin-1 in *Saccharomyces cerevisiae*, J Biol Chem, 286 (2011) 15565-15576.

[2] P. Picotti, H. Lam, D. Campbell, E.W. Deutsch, H. Mirzaei, J. Ranish, B. Domon, R. Aebersold, A database of mass spectrometric assays for the yeast proteome, Nat Methods, 5 (2008) 913-914.

Fig. S1

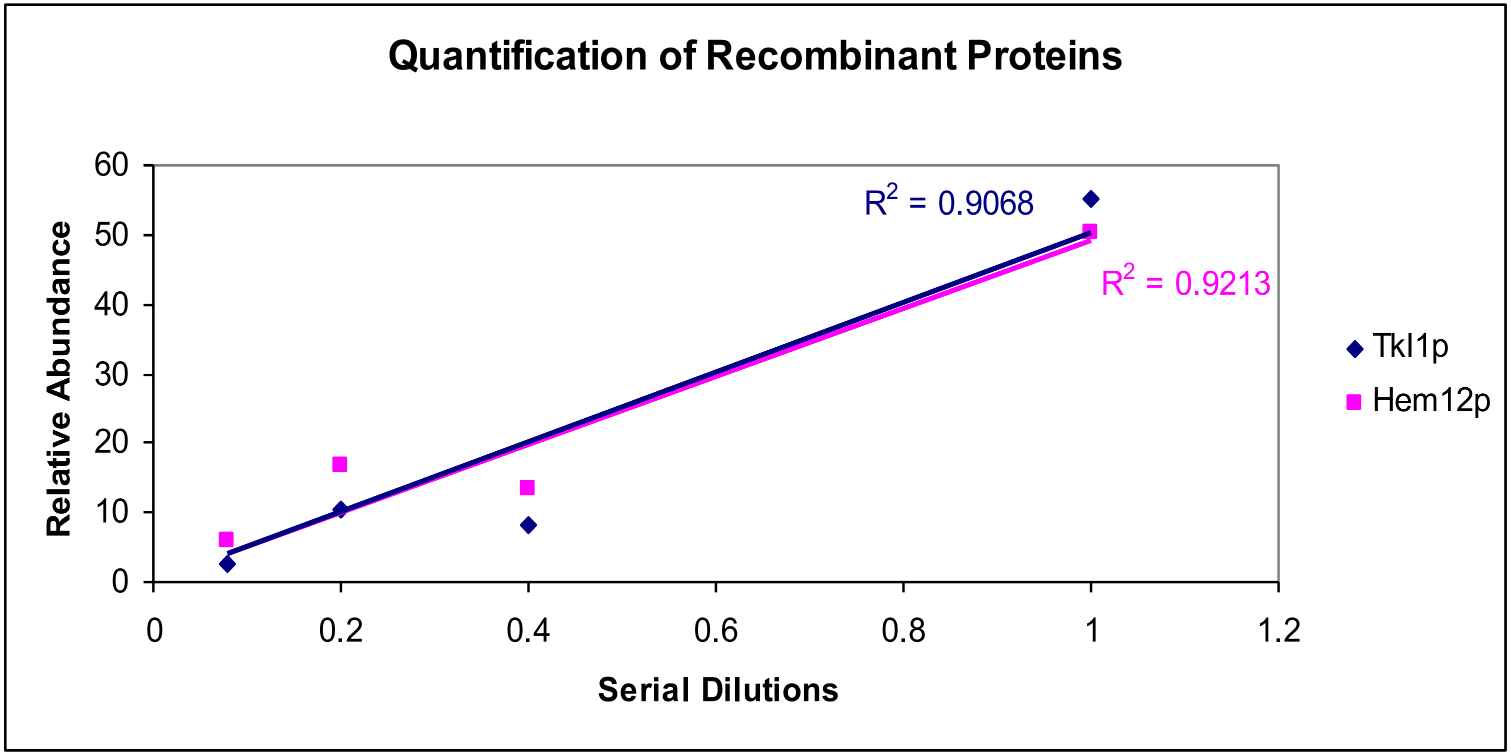

Figure S1. Relative quantification of tryptic digested and serial diluted recombinant proteins analysed in 4000 QTrap (AB Sciex) with label free quantification using Progenesis. Dilution of recombinant proteins followed by tryptic digestion and quantification of peptides detected indicates the detection is proportional to their relative concentration.

Fig. S2

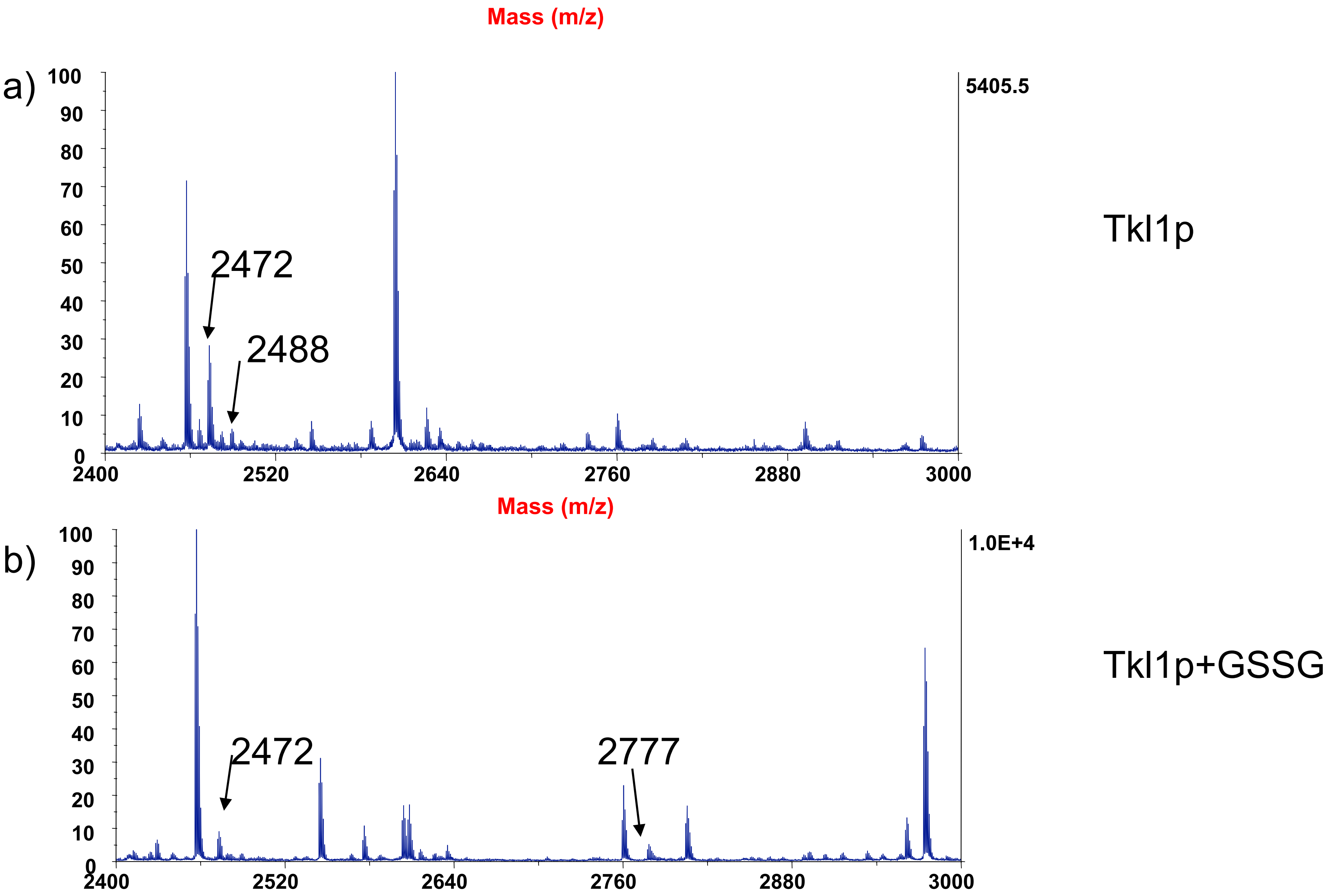

Figure. S2. MALDI-TOF analysis of tryptic peptide (610-632) from a) Tkl1p with reduced (2472 m/z) and sulfenic form (2488 m/z) of Cys622 and b) Tkl1p+GSSG with reduced and glutathionylated form (2777 m/z) of Cys622. Peptides were identified by MALDI-TOF/TOF.
